# Signatures of Value Comparison in Ventral Striatum Neurons

**DOI:** 10.1371/journal.pbio.1002173

**Published:** 2015-06-18

**Authors:** Caleb E. Strait, Brianna J. Sleezer, Benjamin Y. Hayden

**Affiliations:** 1 Department of Brain and Cognitive Sciences and Center for Visual Science, University of Rochester, Rochester, New York, United States of America; 2 Neuroscience Graduate Program, University of Rochester, Rochester, New York, United States of America; Oxford University, UNITED KINGDOM

## Abstract

The ventral striatum (VS), like its cortical afferents, is closely associated with processing of rewards, but the relative contributions of striatal and cortical reward systems remains unclear. Most theories posit distinct roles for these structures, despite their similarities. We compared responses of VS neurons to those of ventromedial prefrontal cortex (vmPFC) Area 14 neurons, recorded in a risky choice task. Five major response patterns observed in vmPFC were also observed in VS: (1) offer value encoding, (2) value difference encoding, (3) preferential encoding of chosen relative to unchosen value, (4) a correlation between residual variance in responses and choices, and (5) prominent encoding of outcomes. We did observe some differences as well; in particular, preferential encoding of the chosen option was stronger and started earlier in VS than in vmPFC. Nonetheless, the close match between vmPFC and VS suggests that cortex and its striatal targets make overlapping contributions to economic choice.

## Abstract 2

A study of single neurons in the ventral striatum reveals signatures of value comparison and selection during a risky choice task, suggesting that the cortex and its striatal targets make overlapping contributions to the choice process. Read the accompanying Primer.

## Author Summary

The neural calculations underlying reward-based choice are closely associated with a network of brain areas including the ventral striatum (VS) and ventromedial prefrontal cortex (vmPFC). Most theories ascribe distinct roles to these two structures during choice, but these differences have yet to be confirmed at the level of single neurons. We compared responses of VS neurons to those of vmPFC neurons recorded in rhesus macaques choosing between potential gambles for water rewards. We found widespread similarities in the way that VS and vmPFC neurons fire during the choice process. Neurons in both areas encoded the value of the offered gamble, the difference in value between offered gambles, and the gamble outcome. Additionally, both areas showed stronger coding for the chosen gamble than for the unchosen one and predicted choice even when we controlled for offer value. Interestingly, preferential encoding of the chosen option was stronger and started earlier in VS than in vmPFC. Nonetheless, similarities between vmPFC and VS suggest that cortex and its striatal targets make overlapping contributions to reward-based choice.

## Introduction

Making beneficial choices about rewarding options is a major function of our brains and is critical for our survival. Consequently, understanding the mechanisms of reward-based choice is a major goal of psychology, microeconomics, animal behavior, and psychiatry [1–7]. Recent empirical and theoretical work has begun to uncover the basic underpinnings of reward-based choice (reviewed in [8–11]). Research into this topic is directly inspired by the success of the perceptual decision-making research program [12,13]. One reason why we currently lack a correspondingly detailed understanding of reward-based choice is that the full set of brain structures involved in this process, and their specific functions, has yet to be established.

In particular, it remains unclear whether reward-based choice takes place in a single core structure that has a dedicated value comparison function, or whether it occurs more broadly, as comparison steps are implemented in unison across different brain regions [14]. Among brain regions associated with reward-based choice, we are particularly interested in the ventral striatum (VS) and the ventromedial prefrontal cortex (vmPFC) [15]. Both regions are associated with option evaluation and with value comparison in neuroimaging and lesion studies [16–24]. On the one hand, this similarity in response properties suggests that they may play similar roles in reward-based choice. On the other hand, much evidence points to distinct roles for the vmPFC and VS. Specifically, VS, like other striatal regions, is generally linked to learning, including habit learning, and to action selection, while vmPFC, like other prefrontal regions, is associated with executive control and flexible, online regulation of behavior [25–40]. Of course, there is a sizable literature on the contributions of ventral striatum to reward-based choice, including action selection [37–40]. These include learning, or action-selection–centered approaches (e.g., actor-critic models, in which VS learns to predict future rewards, while PFC formulates a choice policy designed to maximize reward [41,42]), and gating or modulation theories, wherein the ventral striatum facilitates motor plans by disinhibiting motor plans [43,44]. Indeed, one recent paper found value coding in VS precedes choice but only follows choice in orbitofrontal cortex (OFC; a structure that is adjacent to vmPFC), suggesting that it is VS, and not cortex, that directs the choice [45].

One common trend among these models is that they generally attribute ventral striatum and cortical areas different, and generally complementary, functions. The different roles assigned to cortical and striatal reward signals may reflect true functional differences between these areas, but it is difficult to know for certain without data collected in the same tasks with the same methods in the two areas.

We recently examined the function of Area 14 of the vmPFC in a simple reward-based choice task [15]. We found that neuronal responses encode offers’ subjective values using a single value scale. That is, they integrate across dimensions to form an abstract value variable and then gradually come to encode the value of the chosen—as opposed to unchosen—option. When two offers are made, neurons show opposed tuning for their values—suggestive of a mutual inhibition choice process [15,16,46,47]. Finally, these neurons also showed choice probability correlations, suggesting that their activity may contribute directly to selection [15,48]. We argued that these responses implicate vmPFC in a mutual inhibition process that implements value comparison. On the one hand, vmPFC (possibly along with OFC) may be relatively unique in its role, and other connected brain areas, like VS, dorsolateral prefrontal cortex, and anterior cingulate cortex may play complementary roles less central to choice [8,10,49–53]. On the other hand, such regions may play roles similar to that of vmPFC as part of a larger, multi-site comparison process [14]. Among these areas, we are especially interested in the VS because of the widespread assumption that cortex and striatum have strongly distinct roles in cognition.

The main cells of the striatum are medium spiny neurons, inhibitory (GABA-ergic) cells that receive inputs from cortex and that transmit information to the pallidum [44–56]. The question of just how much interaction there is within the striatum has been one of the most important ones in striatal anatomy and function over the past three decades [57]. Nonetheless, it is very likely that there is at least some within-striatum processing going on [58]. First, there is some (but not decisive [59,60]) evidence for lateral inhibition effects within the striatum [61–69]. The extent of these functional connections is quantifiable in vitro [70,71]. Indeed, some of these connections are reciprocal [58]. There are also more esoteric possibilities for intrastriatal interactions as well, such as nitric oxide communication through gap junctions [72–75]. In any case, there is clear support for the idea of within-striatum processing, supporting the idea that some mutual inhibition may be occurring within the striatum itself.

We found that VS, like vmPFC, represents abstract values and value differences, suggestive of a process of mutual inhibition [15,16,24]. (Note that competitive interaction within the striatum is anatomically plausible [58–60].) We also observed preferential selectivity for chosen, as opposed to unchosen options and choice probability correlates. Both areas encoded outcomes of gambles more strongly than other task variables. Relative to vmPFC, the effects in VS were observed at roughly the same frequency, although they were slightly more common in VS, and preferential representation of the chosen option occurred earlier in time. Aside from these differences, we did not observe any major functional differences in vmPFC and VS response properties. These findings suggest that the basic microcomputations supportive of choice processes can be observed in both cortical and subcortical reward areas. More broadly, they provide tentative support for the idea that choice cannot be localized to one specific region of the brain, but instead reflects the outcome of comparison processes occurring in multiple brain regions.

## Results

**Fig 1:**
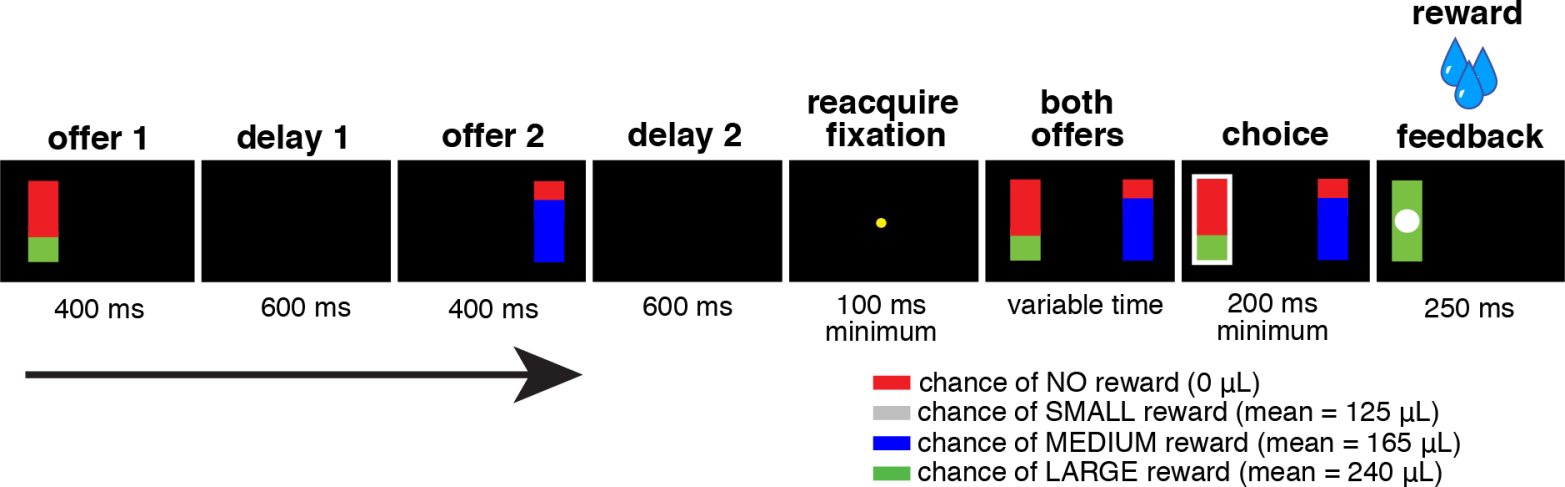
Gambling task timeline. Two potential gambles for water reward were presented each trial. Gambles were represented by a rectangle, some proportion of which was grey, blue, or green, corresponding to a small, medium, or large reward respectively. The size of the grey, blue, or green portion corresponded to the probability that selecting that gamble would lead to the corresponding reward. Offers appeared in a random order one at a time with a one-second offset for 400 ms each. After fixation, both offers reappeared during a decision phase. Rewarded outcomes were accompanied by a white circle in the center of the chosen offer. These data are available in Data S1 on figshare (http://figshare.com/articles/Data_for_Signatures_of_value_comparison_in_ventral_striatum_neurons_/1332487).

Two monkeys performed a two-option gambling task with asynchronous presentation of high-stakes and low-stakes options (see Methods and **Fig 1**). We used this same task previously to delineate the role of vmPFC in reward-based choices [15] (see Methods). One subject (monkey B) was used in the previous study; the other (monkey C) was not. (The choice of recording subjects in both studies was determined solely by the positioning of the chamber, which was done before data collection began.) Behavioral patterns were nearly identical to those observed in the previous study using the same task [15]. Monkeys chose the offer with the higher expected value (i.e., average long-term value associated with the reward) 82.54% of the time. This rate was not significantly different between trials with two offers of the same reward size (82.98%) and trials with two different reward sizes (82.26%; chi-square, X^2^ = 2.1088, *p* = 0.1465). Both subjects were risk-seeking, preferring risky offers to safe offers when both offers had the same expected values (see **S1 Text** for more detail).

### Single Unit Responses

**Fig 2:**
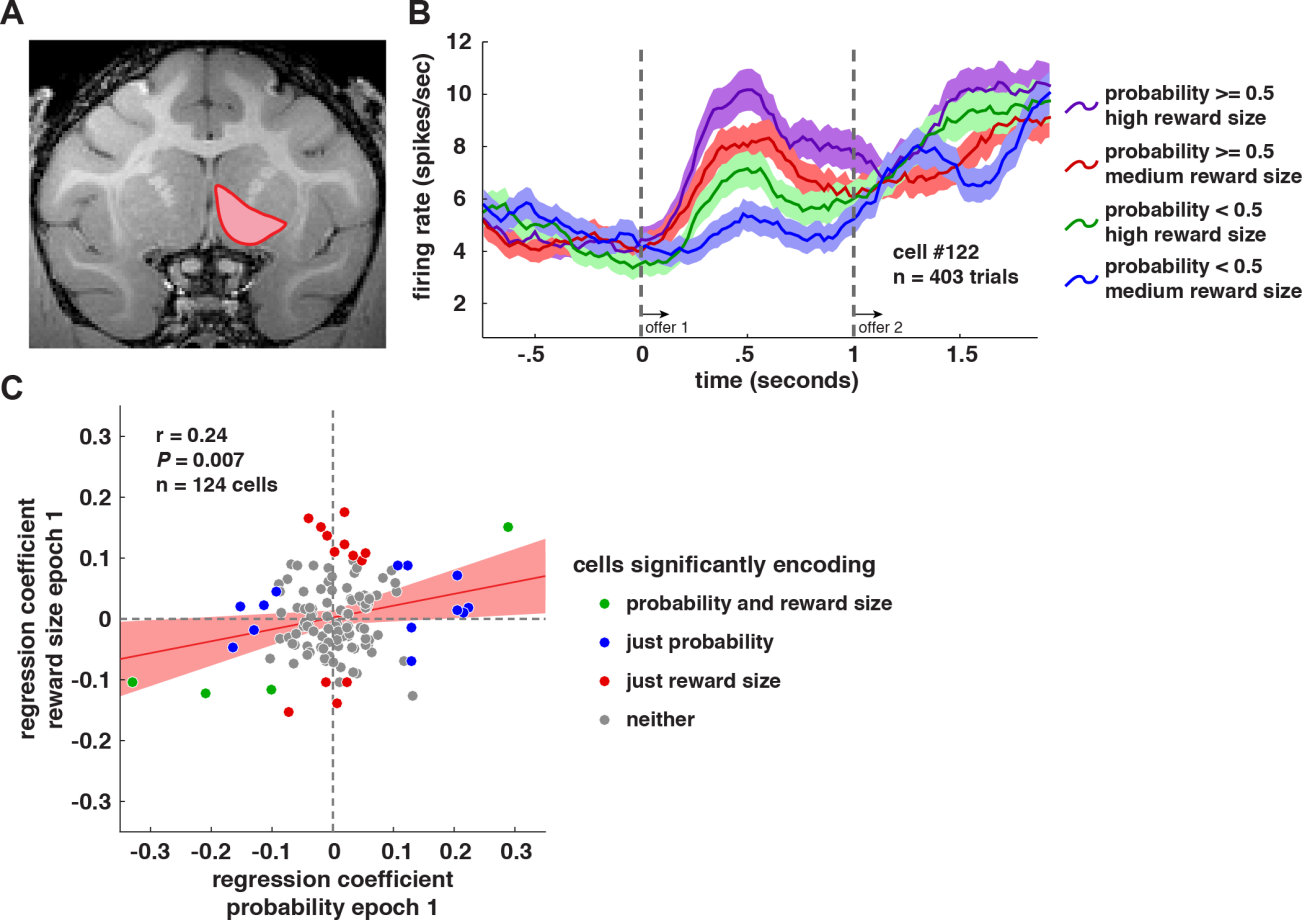
Coding of offer values in VS neurons. **A.** Magnetic resonance image of monkey B. Recordings were made in the nucleus accumbens region of VS (highlighted in red; see S4 Fig for precise demarcation). **B.** Average responses of an example neuron (+/- 1 SEM in firing rate), separated by offer 1 reward size and probability. During epoch 1, this neuron showed higher firing rates for offers with larger reward sizes and probabilities. **C.** Scatter plot of each neuron’s coefficients for tuning for gamble probability (*x*-axis) and gamble reward size (*y*-axis). These coefficients were significantly correlated, consistent with a single value scale coding scheme. A least-squares regression line and confidence intervals are shown in red. Neurons are shown color-coded by regression coefficient *p*-value at α = 0.05. These data are available in Data S1 on figshare (http://figshare.com/articles/Data_for_Signatures_of_value_comparison_in_ventral_striatum_neurons_/1332487).

Our dataset consists of responses from 124 VS neurons (55 neurons in monkey B, 69 neurons in monkey C). Our published vmPFC dataset contains 156 vmPFC neurons recorded in the same task (106 in monkey B, 50 in monkey H; [15]). In this VS study, we recorded an average of 510.2 trials per neuron (range: *n* = 168 to *n* = 813 trials). Neurons were localized to the ventral striatum (Figs **2A** and **S4**). We defined three task epochs for analysis. (To make comparison with our earlier study easier, we use the same epochs and names for epochs we used in our earlier study; [15].) Epoch 1 began with the presentation of offer 1, epoch 2 began with the presentation of offer 2, and epoch 3 began with the gamble outcome. Each epoch lasted 500 ms. We favor a 500 ms time window because (1) it allows us to detect even sluggish responses and (2) by using the same epoch across studies, we reduce the chance of inadvertent p-hacking.

We found that 55.65% of neurons (*n* = 69/124) showed some sensitivity to task events, as indicated by individual cell ANOVAs of firing rate against epoch for the three task epochs and a fourth 500 ms inter-trial epoch (*p <* 0.0001, binomial test). For comparison, we found that 46.15% of neurons (*n* = 72/156) in vmPFC showed some sensitivity to task events. These results indicate that VS neurons are slightly, but not significantly, more likely to respond to the task than vmPFC neurons (chi-square test, X^2^ = 2.4896, *p* = 0.1146). All proportions refer to all recorded neurons, not just ones that produced a significant response modulation.

### Neurons Encode Offer Value in an Abstract Format

We first examined coding of economic variables (i.e., probability and reward size) in the first offer epoch (epoch 1). **Fig 2B** shows responses of an example neuron to offers separated by their probability and reward size. In epoch 1 this neuron’s firing rates encoded the probability of offer 1 (linear regression; *β* = 0.1602, *p* = 0.0013) and the reward size of offer 1 (*β* = 0.1667, *p* = 0.0008). We found that the firing rates of 14.5% of cells (*n* = 18/124) were correlated with (and thus, in our parlance, “encoded”) the probability of winning (linear regression, α = 0.05; **Table 1**). This proportion of encoding neurons is much greater than expected by chance, and so is unlikely to reflect random noise (binomial test, *p <* 0.0001). In this first epoch, the same proportion of neurons (14.5%) encoded the potential reward size available (i.e., gamble stakes). These proportions are similar to but slightly higher than the analogous proportions observed in vmPFC neurons (during epoch 1, 7.7% of vmPFC neurons encoded probability and 11.5% encoded reward size; [15]). Note that safe offers, which occurred on 12.5% of trials, have a fixed 100% reward probability and a relatively small reward. Therefore they make high probability offers more likely to have small reward sizes than not. This introduces a negative correlation between reward size and probability, and as a result, trials with safe offers are excluded from this analysis.

**Table 1:** Neurons encode offer value in an abstract format.

	ventral striatum	ventromedial prefrontal cortex
probability of winning	14.5% of cells (18/124)	7.7% of cells (12/156)
	*p <* 0.0001	*p* = 0.05
reward size	14.5% of cells (18/124)	11.5% of cells (18/156)
	*p <* 0.0001	*p* = 0.0003

Proportions of neurons in ventral striatum and ventromedial prefrontal cortex significantly encoding each value dimension in epoch 1 (0–500 ms after offer 1 presentation). These data are available in Data S1 on figshare (http://figshare.com/articles/Data_for_Signatures_of_value_comparison_in_ventral_striatum_neurons_/1332487).

We asked whether VS neurons carry an integrated reward signal, as is the case in vmPFC [15]. The alternative is that they preferentially encode either probability or reward, or even both orthogonally; such coding schemes may be used in area 13 of OFC [53,76]. To address this question, we compared regression coefficients for firing rate versus probability with coefficients from the regression of firing rate versus reward size (again, limiting ourselves to epoch 1). If neurons encode an abstract value form of offer value [8,77] then their separately calculated regression coefficients for probability and reward size should themselves be positively correlated [15]. Such abstract value coding would be consistent with the use of a single value scale to encode reward amount. Indeed, we found a significant positive correlation between these coefficients (*R* = 0.24, *p* = 0.007; **Fig 2C**). These data are consistent with the idea that the ensemble of VS neurons represents value in an abstract format. Moreover, they mirror those found in vmPFC (*R* = 0.25, *p* = 0.0023; [17]), and suggest that abstract reward value representation occurs in both cortex and ventral striatum [8,27,78].

Do VS neurons have qualitatively different response latencies for value coding than neurons in vmPFC? First, we separated trials into high or low offer 1 value categories. Then, using a sliding *t* test, we found the first 20 ms period after offer 1 presentation where a *t* test on firing rates in those two sets of trials was significant at *p <* 0.05. This process found a significant difference sometime in the 500 ms after offer 1 presentation for 23/124 VS cells and 17/156 VM cells. We found no significant difference between these VS cell response latencies (mean: 78.52 ms) and vmPFC cell latencies: (mean: 75.73 ms) for offer 1 value coding after offer 1 presentation (*t* test, t(38) = 0.2588, *p* = 0.7972).

### Antagonistic Coding of Competing Offer Values

**Fig 3:**
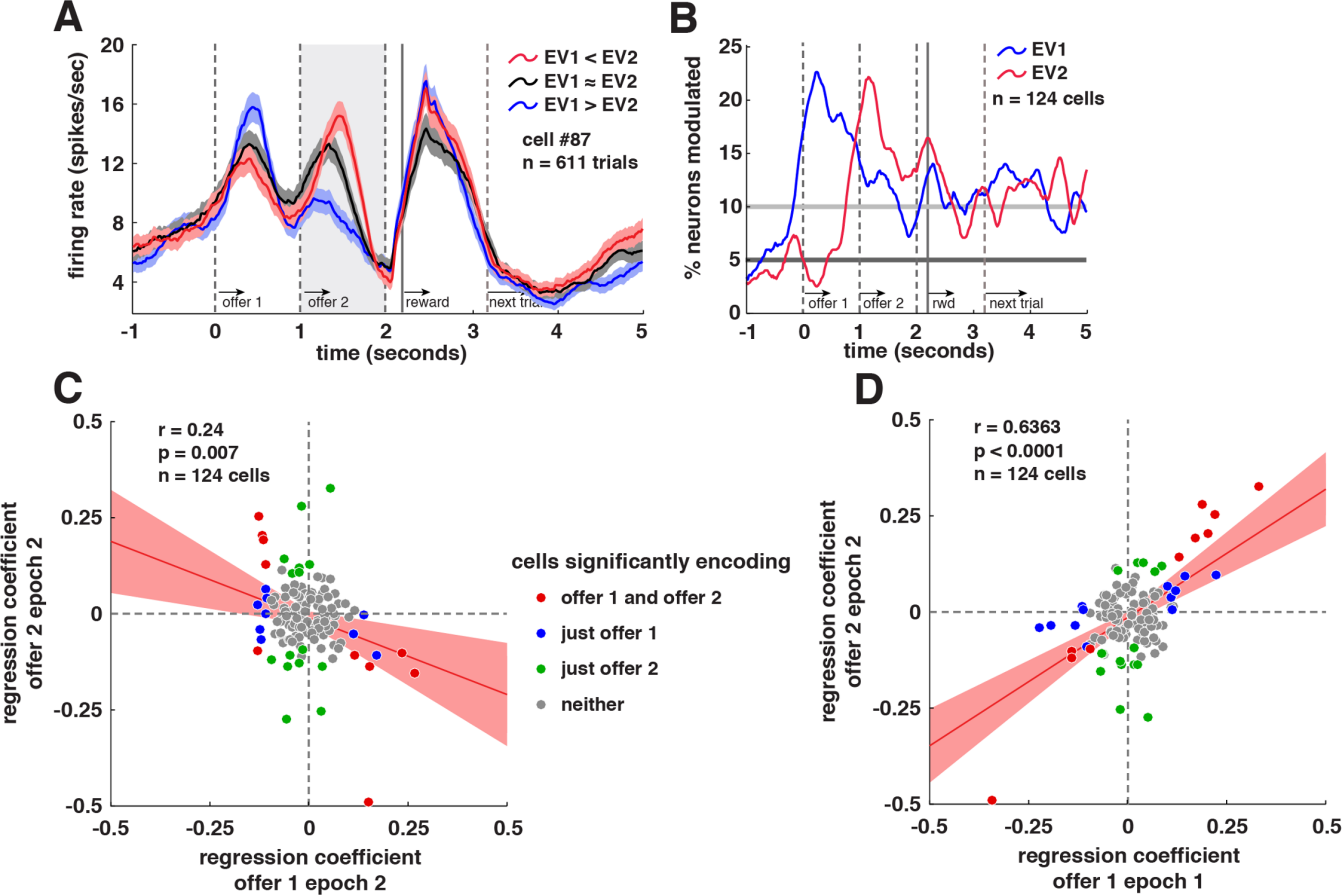
VS neuron activity related to comparison and choice. **A.** Average responses of an example neuron (+/- 1 SEM in firing rate), separated by binned (lowest 33.3%, middle 33.3%, highest 33.3%) expected value difference between offer values (offer value 1 minus offer value 2). During the second offer presentation, this neuron fired more when offer value 2 was greater than offer value 1 (red), and less when offer value 1 was greater than offer value 2 (blue). **B.** Plot of the proportion of neurons that show a significant correlation between neural firing rate and the value of the 1st (blue) and 2nd (red) offers (500 ms sliding boxcar). Horizontal lines show when the proportion of cells shown reaches significance (dark gray: 5%; light gray: binomial test at α = .05). **C.** Scatter plot of each neuron’s coefficients for tuning for offer value 1 (*x*-axis) and for offer value 2 (*y*-axis), both in epoch 2. Least-squares regression line and confidence intervals are shown in red. Neurons are shown color-coded by regression coefficient *p*-value at α = 0.05. **D.** Scatter plot of each neuron’s coefficients for tuning for offer value 1 in epoch 1 (*x*-axis) and for offer value 2 in epoch 2 (*y*-axis). Neurons are shown color-coded by regression coefficient *p*-value at α = 0.05. These data are available in Data S1 on figshare (http://figshare.com/articles/Data_for_Signatures_of_value_comparison_in_ventral_striatum_neurons_/1332487).

In order to look for offer value signals in VS neurons, we operationalized an offer’s value as its gamble’s expected value, that is, its reward magnitude multiplied by its reward probability. **Fig 3A** shows responses of an example neuron to offers separated by their relative expected values (value of offer 1 minus value of offer 2). Its firing rates encoded the expected value of offer 1 in epoch 1 (linear regression; *β* = 0.1705, *p <* 0.0001) and in epoch 2 (*β* = -0.0985, *p* = 0.0149). The sign flip indicates that the direction of tuning for offer 1 was reversed for the second epoch. This neuron also encoded the expected value of offer 2 in epoch 2 (*β* = 0.1698, *p <* 0.0001), meaning that during epoch 2 it coded both values simultaneously. This propensity to code two values simultaneously was observed across the population (**Fig 3B**). In epoch 2, significant proportions of neurons encoded both offer value 1 (*n* = 16/124, 12.9%) and offer value 2 (*n* = 27/124, 21.8%). These value signals are robust to time window changes, for example, +/- ~100 ms around the responses seen in Fig 3B.

In our example neuron, tuning directions for expected values 1 and 2 have opposed signs. This anti-correlation is consistent with antagonistic coding of these offers, i.e., representations of the two values interact competitively to influence the firing rate of this neuron (cf. [16]). This antagonistic pattern is observed at the population level as well. Overall regression coefficients for offer value 1 in epoch 2 are anti-correlated with coefficients for offer value 2 in the same epoch (*R* = -0.2919, *p* = 0.0010, **Fig 3C**). To match the criteria used in the above analyses, this analysis does not include trials with safe options; however, if we repeat the analysis with the safe offer trials as well, we find the same anti-correlation (*R* = -0.2766, *p* = 0.0019). This finding of antagonistic coding may be a signature of comparison through mutual inhibition and is also observed, at a slightly weaker strength, in vmPFC neurons (*R* = -0.218, *p* = 0.006; [15]).

We next looked at response latencies for antagonistic value coding during epoch 2 by separating trials by which of the two offers had a higher value. Using a sliding *t* test, we found the first 20 ms period after offer 2 presentation in which a *t* test on firing rates in those two sets of trials was significant at *p <* 0.05. We found a significant difference sometime in the 500 ms after offer 2 presentation for 22/124 VS cells and 15/156 VM cells. We found no significant difference between VS cell response latencies (mean: 119.35 ms) and vmPFC cell latencies: (mean: 92.42 ms) for antagonistic value coding after offer 2 presentation (*t* test, t(35) = 0.5014, *p* = 0.6192).

We found 14 fast-spiking interneurons (FSIs) and 66 medium spiny neurons (MSNs) in our 124 neuron population, using waveform criteria as delineated by Jin et al. [79]. Four of the fourteen FSIs (28.6%) and 10 of the 66 MSNs (15.2%) significantly encode the difference between the offered values during epoch 2 (correlation, *p* < 0.05). Although small, these proportions are both greater than what would be expected by chance (binomial tests, FSIs: *p* = 0.0004; MSNs: *p* = 0.0004). The ratio of FSIs that show antagonistic coding is not significantly different from that of the MSNs (chi-square, X^2^ = 1.4408, *p* = 0.2300).

The data presented so far are consistent with the idea that VS contains two distinct memory buffers for reward value, one for currently presented options and the other for previously presented options stored in working memory (cf. [80]). To further test this idea, we examined the relationship between a vector of regression coefficients for option 1 in epoch 1 and option 2 in epoch 2 for all cells. We found a significant positive correlation between these vectors (*R* = 0.6363, *p <* 0.0001; see **Fig 3D**). This suggests that whatever effect a larger offer 1 had on firing rates during epoch 1 in each neuron (excitatory or suppressive), the same effect was observed for those neurons to a larger offer 2 in epoch 2. This finding suggests that VS neurons use a single coding framework consistently across time to code the currently offered option (cf. [24]). This consequently suggests that neurons do not use a single format to represent a single option’s value over the course of a trial. Instead, the format used is different in the context in which the option is currently offered and in the context in which the option was offered in the past and presumably remembered. This context-dependent coding pattern for offered options across the two epochs was also observed in vmPFC neurons [15].

### Neurons Are Tuned for Chosen Offer Value, but Not Unchosen Offer Value

**Fig 4:**
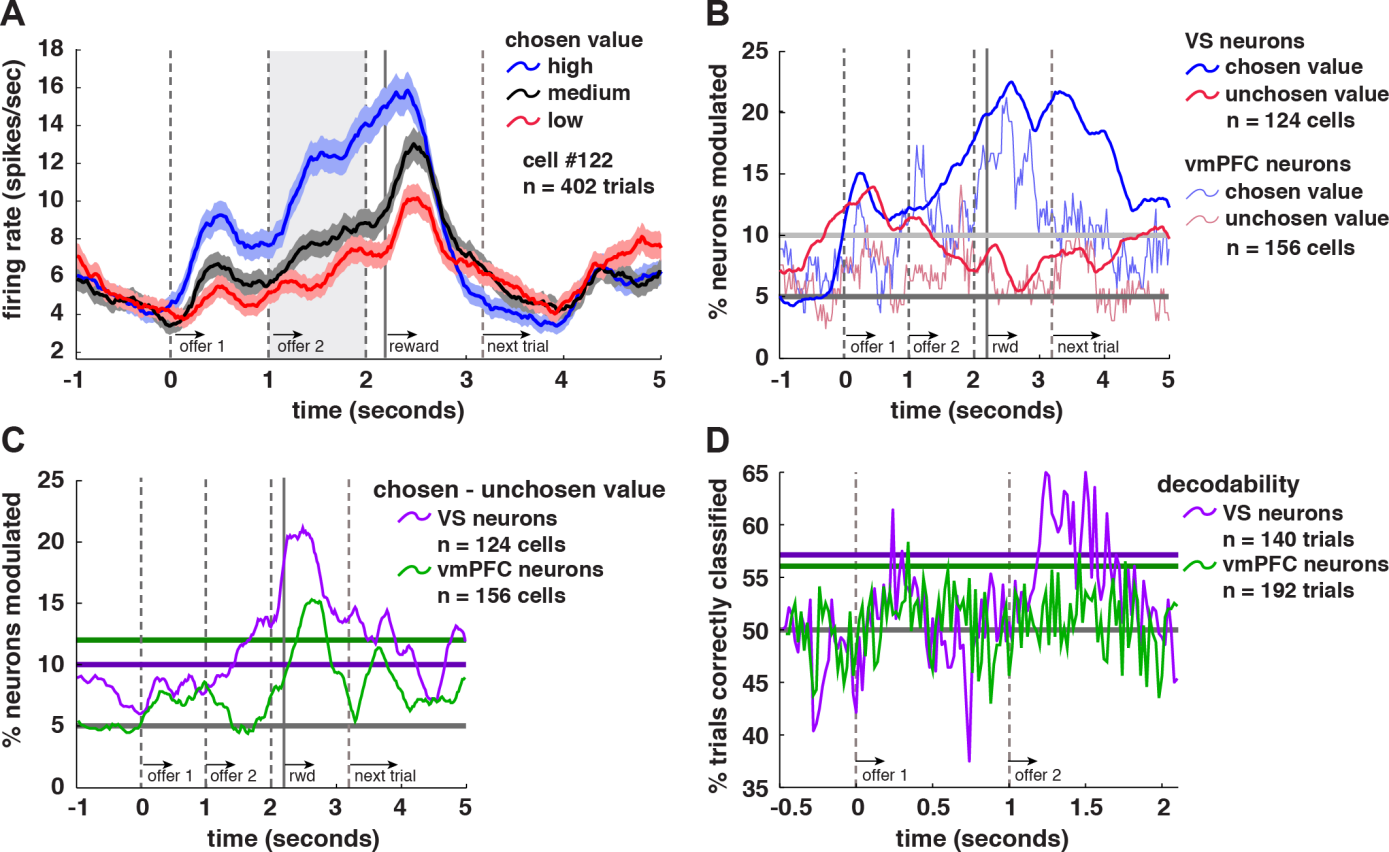
VS neurons come to code chosen value. **A.** Average responses of an example neuron (+/- 1 SEM in firing rate), separated by binned expected value of the chosen offer. This neuron showed higher firing rates when the value of the eventually chosen offer was greater than average (blue), and lower firing when the chosen offer value was lower than average (red) starting in epoch 1 (knowing offer 1 gives the monkey partial information as to his eventual chosen offer) and extending into epochs 2 and 3. **B.** Plot of proportion of neurons that show a significant correlation between firing rates and the value of the chosen (VS: dark blue line; vmPFC: light blue line) and unchosen (VS: dark red line; vmPFC: light red line) offers (500 ms sliding boxcar). Note that each point on a sliding boxcar plot is derived from a 500 ms bin beginning at that point, so it is feasible for significant encoding to arise before offer presentation on the plot. Horizontal lines show when the proportion of cells shown reaches significance (dark gray: 5%; light gray: binomial test at α = .05 on VS dataset). vmPFC data from [15]. **C**. Proportion of cells whose firing rates significantly encoded value difference (chosen value-unchosen value). The horizontal lines show when the proportion of cells shown reaches significance (gray: 5%; purple: binomial test at α = .05 on VS dataset; green: binomial test at α = .05 on vmPFC dataset). **D.** Decodability of chosen offer in a 500 ms sliding boxcar for VS neurons (purple) and vmPFC neurons (green; see Methods). The horizontal lines show when the proportion of trials correctly classified reach significance (gray: 50%; purple: binomial test at α = .05 on VS dataset; green: binomial test at α = .05 on vmPFC dataset). These data are available in Data S1 (VS) and Data S2 on figshare (vmPFC; http://figshare.com/articles/Data_for_Signatures_of_value_comparison_in_ventral_striatum_neurons_/1332487).

**Fig 5:**
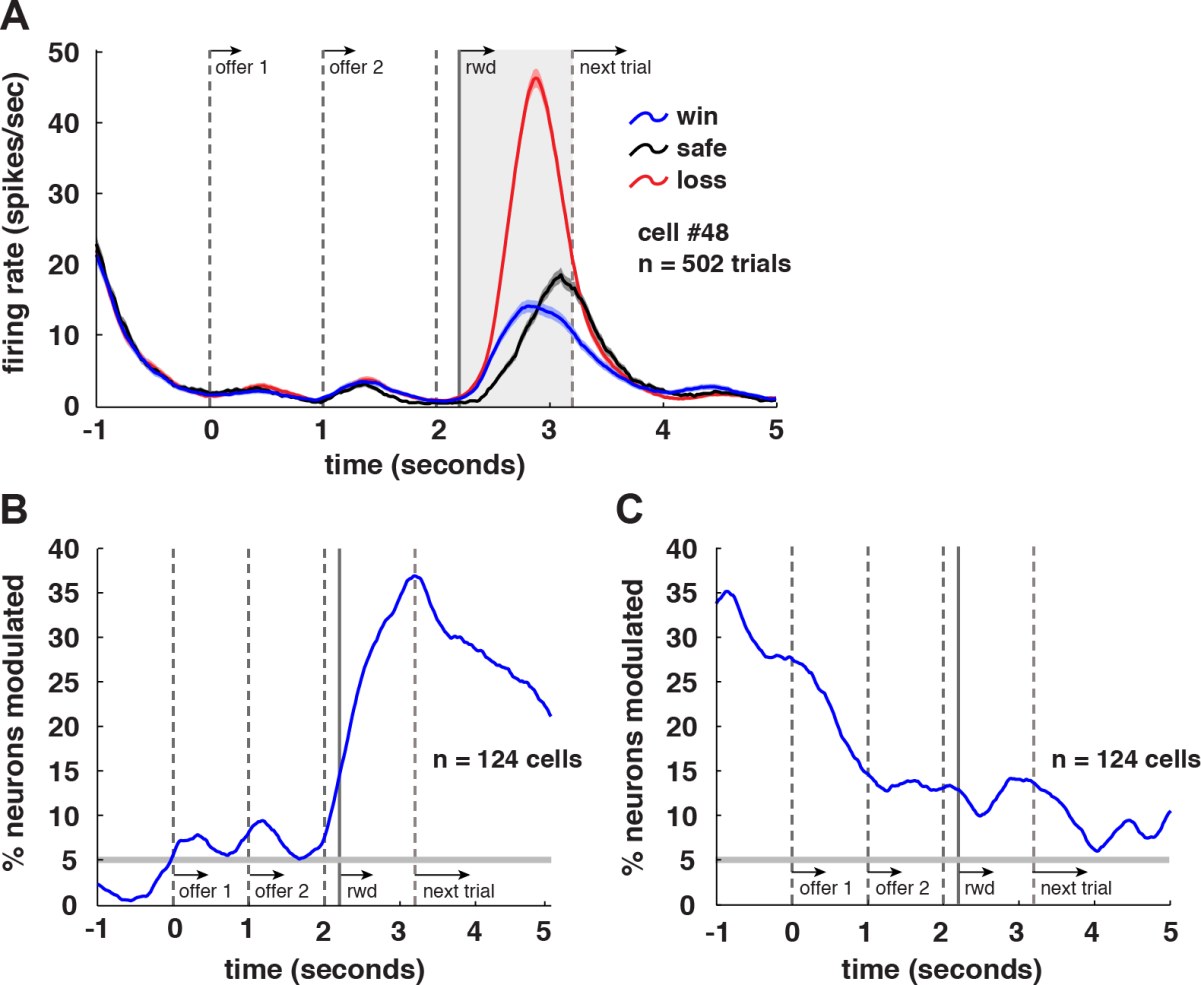
Coding of outcomes in VS neurons. **A.** Average responses (+/- 1 SEM in spks/s) of an example neuron to task events, separated by gamble outcome. This neuron showed a negative tuning for outcome during epoch 3 (shaded area). **B.** Plot of proportion of neurons significantly tuned for gamble outcomes over time using a 500 ms sliding window. **C.** Same data as in B, but with tuning for outcome on previous trial instead of on current trial. Influence of the previous trial’s outcome was strong and lasted throughout the current trial. These data are available in Data S1 on figshare (http://figshare.com/articles/Data_for_Signatures_of_value_comparison_in_ventral_striatum_neurons_/1332487).

After determining that neurons in VS encode the values of both offers simultaneously and antagonistically in epoch 2, we next examined whether they preferentially signal the chosen one. **Fig 4A** shows responses of an example neuron to offers separated by the expected value of the chosen offer. Its firing rates encoded the expected value of the chosen offer in epoch 1 (knowing offer 1 gives the monkey partial information as to his eventual chosen offer; *β* = 0.2298, *p <* 0.0001, linear regression) and on into epoch 2 (*β* = 0.2765, *p <* 0.0001) and epoch 3 (*β* = 0.2420, *p <* 0.0001). **Fig 4B** shows the proportion of neurons in each dataset whose activity is significantly modulated by chosen offer values (VS: dark blue line; vmPFC: light blue line) and by unchosen offer values (VS: dark red line; vmPFC: light red line) in a sliding 500 ms time window. Note that **Fig 5A and 5B** both show a peak during epoch 3 that is even larger than the peak in epoch 2 because the value of the chosen offer was highly correlated with the value of the outcome, the coding of which was stronger than other effects; see below.

We found the same coding frequency for the value of the chosen and unchosen offers during epoch 1 (12.9% of cells for both chosen and unchosen options, *n* = 16/124) suggesting that VS does not distinguish chosen from unchosen options at this point, or if it does so, does it too weakly to detect with this analysis. In the first 500 ms of epoch 2, we again found no difference in coding of chosen and unchosen offers (9.7% coded chosen and 12.9% of cells coded unchosen), but by the end of this epoch (the last 500 ms), we saw the gradual emergence of a preference for a chosen offer. Specifically, we saw stronger coding for chosen offers than for unchosen offers (15.3% and 6.5% of cells, respectively; 15.3% is significantly more than chance, *p <* 0.0001; 6.5% is not, *p* = 0.1689). Note that this change in coding frequency, 6.5% to 15.3%, is itself significant, χ^2^ = 16.168, *p <* 0.0001.

As shown in **Fig 4C**, we observed a gradual increase in the proportion of cells whose firing rates significantly encoded this difference (between chosen value and unchosen value). The horizontal lines show when the proportion of cells shown reaches significance (gray: 5%; purple: binomial test at α = 0.05 on VS dataset; green: binomial test at α = 0.05 on vmPFC dataset). The proportion of VS neurons’ first significant bin was 1.42 s after offer 1 presentation, while the proportion of VM neurons’ first significant bin was 2.39 s after offer 1 presentation. These results suggest that VS has access to information about the choice process before vmPFC. Indeed, in VS the preferential coding occurred before the saccade that implements the choice, but this was not the case vmPFC.

Another way to look at the timing of choice-related signals is to look at decodability of chosen offer as a function of time in the trial. In other words, we examine how accurate an ideal observing decoder would be at decoding eventual choice (offer 1 or offer 2) from firing rates as the trial progresses (see **Methods**). **Fig 4D** shows decodability of chosen offer in a 500 ms sliding boxcar for VS neurons (purple) and vmPFC neurons (green). The horizontal lines show when the proportion of trials correctly classified reach significance (gray: 50%; purple: binomial test at α = .05 on VS dataset; green: binomial test at α = .05 on vmPFC dataset). Both VS and vmPFC cells showed peaks of significant choice decodability during epoch 1 (VS onset: 240 ms after offer 1 presentation; vmPFC onset 316 ms) and again in epoch 2 (VS onset: 180 ms after offer 2 presentation; vmPFC onset 440 ms). It is important to note that peaks of significant choice decodability are quite transient in vmPFC during both epochs and in VS during epoch 1, and therefore may be due to statistical noise. These results suggest that choices may be more quickly and more reliably decodable in VS than in vmPFC. Note that a more sensitive test of choice-related variance did show significant encoding of choice prior to the choice saccade in vmPFC ([15]; see below).

### Variability in Firing Rates Predicts Choice

To further investigate the connection between neural activity in VS and choice, we made a calculation similar to a choice probability [81]. For each neuron, we first regressed firing rate in epoch 1 onto offer 1 value, probability, and reward size, and determined the residuals. We then examined whether the values of the residuals from this regression predicted choice (offer 1 versus offer 2) for each neuron. To associate the residuals with choice, we simply ran a binomial regression on choice as a function of the (continuous) residual variable. (We confirmed that a simple correlation test produces similar results.) In other words, we computed the residual variance in firing rate after accounting for the factors that influence value. We found a significant relationship between residual firing rate variance and choice in 8.87% of cells (*n* = 11/124), which is more than is expected by chance (*p* = 0.0218, binomial test). Given that offer 2 has yet to be presented and the choice is not yet made, it may seem odd for choice selectivity to be present in epoch 1. However, we believe this result is expected in the case that response to offer 1 represents value and that value representation in turn influences choice. Likewise, residual variation in firing rate in response to offer value 2 during epoch 2 predicted choice in 12.90% of cells (*n* = 16/124, *p <* 0.0001, binomial test). Correlations between residual firing rate variance and choice following the second offer reveal (mean absolute value correlation coefficient: 0.0629) were stronger than following the first (mean absolute value correlation coefficient: 0.0456; *t* test of correlation coefficients between residual firing rate variance and choice; t(246) = 2.40, *p* = 0.0171). This result is consistent with the idea that the population of VS neurons gradually comes to encode the chosen offer value more than the unchosen offer value as the decision emerges (see **Fig 4B and 4C**). In our earlier study, we also observed choice probability correlates in vmPFC; the preponderance of the effects was similar in both cases [15]. These shared patterns suggest that fluctuations in VS responses, like those in vmPFC, relate, however indirectly, to ongoing choice processes in a similar way.

### Neurons in VS Encode Outcomes of Gamble Choices

Outcome monitoring is a prominent aspect of vmPFC responses [15]. **Fig 5A** shows responses of an example VS neuron with trials separated by gamble outcome. This neuron encoded received reward size in epoch 3 (*R* = -0.8402, *p <* 0.0001, linear regression). We observed a significant encoding of gamble outcome in 33.9% of cells (*n* = 42/124; **Fig 5B**). Of these cells, 50% (*n* = 21/42) showed negative tuning, while the other 50% showed positive tuning. Outcome coding continued across the delay between trials, and previous trial outcome was a major influence on firing rates during both epochs 1 (28.2% of cells, *n* = 35/124) and 2 (16.1% of cells, *n* = 20/124, *p <* 0.0001; **Fig 5C**). Previous trial outcome even influenced responses during the current trial’s outcome epoch in 12.9% of cells (*n* = 16/124).

Above, we reported that coding for offer values 1 and 2 use a single value scale coding format. We next looked at whether the coding format for outcome was similar to that of the coding format for offer value 1 and 2. We did so by comparing tuning profiles for outcome and offer value 1. In particular, we asked whether regression coefficients for offer value 1 in epoch 1 were correlated with regression coefficients for received reward size in epoch 3. Alternatively, these coefficients could be uncorrelated, which would indicate that neurons that fire preferentially for larger offer 1 values are not also the same neurons that fire preferentially for large outcomes. We found a significant correlation between these regression coefficients (*R* = 0.2712, *p* = 0.0023). This suggests that VS neurons use a single, or at least similar, coding scheme to represent offer values and represent outcomes. This finding matches that observed in vmPFC as well (*R* = 0.22, *p* = 0.0054; [15]).

Dopamine neurons do not provide a labeled line representation of reward size; instead their reward encoding is normalized by reward prediction (i.e., it is a reward prediction error [82]). Despite prominent dopaminergic inputs to VS, previous investigations into reward prediction error coding in VS neurons have had a mixture of positive [83] and negative [84] results. We performed a stepwise regression to determine whether, after accounting for reward size (first step of the regression), post-outcome responses in VS are related to probability of that reward (second step). Because many neurons have negative tuning, we flipped the values for neurons that had negative individual tuning profiles, as measured by regression coefficient, whether their regression coefficients were significant or not.

Starting with just risky trials (i.e., with no small, safe offers), the gamble outcome regressor met the criteria for model inclusion (*β* = 0.2017, *p <* 0.0001), but the reward probability of the chosen offer did not (*β* = -0.0082, *p* = 0.6926). We then repeated these analyses for the medium- and high-reward size trials separately. We find similar results when examining only trials in which a medium-reward option was chosen (gamble outcome: *β* = 0.2362, *p <* 0.0001; chosen option reward probability: *β* = -0.0140, *p* = 0.6743) and when examining only trials in which a high-reward option was chosen (gamble outcome: *β* = 0.2224, *p <* 0.0001; chosen option reward probability: *β* = -0.0460, *p* = 0.0806). This finding indicates that pure outcome is a better descriptor of VS outcome-related responses in this task than reward prediction error. These response patterns align VS with vmPFC in the same task, and distinguish them from dACC in a similar (albeit not identical) task [85].

## Discussion

Here we examined the responses of neurons in VS during a gambling choice task we previously used to study the function of vmPFC [15]. We wondered whether these two areas have similar or distinct contributions to choice. We found that task-related response properties of VS neurons are strikingly similar to those in vmPFC. Specifically, we found that neurons in VS show five major response patterns. First, responses to offered gambles encode offer value (i.e., they signaled expected value, not probability and stakes separately). Second, neurons code the difference in value of the two offers (i.e., tuning for the two offers is antagonistic), suggesting a mutual inhibition or tug-of-war-like process. Third, neurons initially signal the values of both options and then gradually come to signal chosen values and not unchosen values. Fourth, residual variation in firing rate after regressing out value coding signals predicts choices. Fifth, neurons show prominent outcome-related responses. We did find some differences: there is clearer evidence that choice-related signals precede choice in VS than in vmPFC (although these signals still reach significance before choice in vmPFC by some measures [15]). Overall, however, the strong overlap in functions of VS and vmPFC suggests that these two regions have similar functions, at least in the context of a straightforward economic choice task.

We hypothesized that neurons in VS participate in the choice process and that they do so as part of an anatomically distributed mutual inhibition process. Specifically, following Hunt and Behrens (2012), we hypothesized that value representations of the two offers in VS compete for control of neuronal activity [15,16]. This hypothesis can be contrasted with two major alternative hypotheses: (1) that choice occurs through a horse-race type process, in which there is no competition and thus no mutual inhibition, and (2) that choice does not occur in this brain region. If our hypothesis is correct, then firing rates should reflect this competition. Specifically, firing rates in neurons that represent values of the offers should be antagonistically affected by those two values (negative correlation between regression coefficients), and not additively (positive correlation) or orthogonally (no correlation), nor should separate sets of neurons represent the values of each option. Thus, we predict that firing rates should reflect the difference in values of the two offers. Our results support this hypothesis: at the population level, regression coefficients for offer 1 value are anti-correlated with regression coefficients for offer 2 value, suggesting a tendency towards antagonistic value coding.

One alternative possibility is that the VS contains two groups of neurons whose firing rates represent either the value of offer 1 or of offer 2, and that they do not respond to the other offer. This situation would be consistent with both of our alternative hypotheses. Fortunately, we can test for this possibility by comparing the absolute values of the correlation coefficients. We find that these are significantly positively correlated, supporting the idea that neurons are drawn from a single, competitively tuned population and inconsistent with the idea that they are drawn from two different populations. In other words, it does not appear to be the case that individual neurons are specialized for one of the two offers; instead, the competition takes place either in the neurons themselves, or in their inputs.

At the least, we show that vmPFC and its striatal target VS both carry three classes of signals related to choices: choice inputs, correlates of middle stages of choice, and choice outputs. These matching patterns of results are consistent with the idea that vmPFC and VS play fundamentally similar, rather than contrasting, roles in choice. However, the existence of these signals does not prove that choices occur within both (or even either) area; one or both may receive copies of this information from other regions. Nonetheless, these data can exclude the hypothesis that choice occurs and is complete prior to information entering into VS. Another possibility is that VS, and not vmPFC, is the site at which comparison occurs. Indeed, one recent paper reported effects consistent with this hypothesis in rats [45]. Future work will be needed to resolve this question.

The similarity between VS responses and those in vmPFC [15] and even OFC [77] may not appear surprising, given that OFC and vmPFC are two major cortical afferents to the region of VS in which we recorded [56]. On the other hand, most theories emphasize the contribution of VS to learning and other processes distal to the evaluation and comparison processes that directly implement reward-based choices [25–32]. In contrast, neural studies of choice processes generally focus on the cerebral cortex. The present results suggest that this view is too narrow, and that VS, like its cortical afferents, may participate directly in computations that are critical for reward-based decisions. In any case, the present results do not imply that prominent accounts of VS function in control of learning are incorrect; quite the contrary, we suspect that cortical regions may have some of the same functions generally ascribed to striatum [86]. Indeed, our task expressly minimizes the importance of learning in its design.

Previous studies of ventral striatum function in a reward-based choice context have generally attributed ventral striatum and cortical areas different, and generally complementary, functions, such as actor-critic models [41] or gating/modulation theories [43,44]. Several research groups have shown that neurons in the dorsal striatum (both caudate and putamen) respond differently depending upon the reward expected from an action, suggesting they encode action values [30,87–89]. These neurons may directly influence choice by providing a bias signal over specific actions [37–40,90,91]. The present results are consistent with idea that VS neurons also encode action value, although they suggest it participates in comparison as well. This comparison process may contribute to the evaluation process critical in actor-critic and actor-director-critic models [29]. These models see the job of the VS to calculate the value associated with actions or abstract choice states and to drive learning accordingly. Interestingly, Samejima et al. found a conspicuous absence of value difference encoding in the dorsal striatum. These results suggest that the dorsal striatum may play a role in reward-based choice, but are generally silent on the question of the function of VS.

In one recent study using a delay discounting task, Cai, Kim, and Lee [27] found prominent value sum signals in VS neurons (20% of cells) and no significant encoding of value difference (5% of cells, the number expected by chance). Nor did they find significant chosen value signals in VS. From these results, they concluded that VS participates in signaling task state but does not contribute to value comparison. We find the results of the two studies to be strikingly different. We suspect that the difference is most likely due to differences in task design—specifically, the use of asynchronous presentation in our task. Hunt and colleagues have demonstrated that asynchronous and simultaneous presentation of offers in reward-based choice task can lead to differential involvement of different structures [46]. If so, by using an asynchronous presentation, we may have uncovered a comparison role that was masked by the task design of this earlier study.

Despite our findings, we are reluctant to abandon localizationism. In regards to value comparison, Rushworth and colleagues have provided some evidence that lateral structures in the OFC, for example, do not participate in comparison, but instead mediate it [10,51,92]. Likewise, Wilson et al. have argued that OFC participates in state signaling but does not directly implement evaluation and comparison processes [93]. Meanwhile, we have shown some evidence that dorsal anterior cingulate cortex (dACC), another reward region that provides inputs to ventral striatum, may not contribute directly to choice, at least under somewhat different choice conditions [50]. Around the time of choice, neurons in dACC carry signals that depend on outcomes of decisions, but not related to value comparison per se. These findings are consistent with other ideas linking dACC to regulation of strategic adjustments and executive control. Further afield, posterior cingulate cortex (PCC) shows blood-oxygen-level dependent correlates of value and salience, but does not appear to implement choice, either [94,95]. Instead, it seems to detect long-term changes that necessitate deeper strategic shifts, including implementation of long-term learning [96–98].

Our data do raise the possibility that multiple brain regions perform similar computations at roughly the same time. If so, then how does the output system—the motor system—adjudicate between competing decisions in order to select the single best course of action? Our data do not provide much guidance on this topic although we hope to pursue this area in the future. Our best guess is that the brain weights different systems based on reinforcement learning principles [11,99–101].

One result that surprised us is the lack of prominent reward prediction error (RPE) signals in VS neurons. This finding differentiates VS from its dopamine inputs, which show clear and prominent RPE signals [82]. One possibility is that, unlike dopamine neurons, VS only carries RPE signals when they lead to adjustments, learning, or changes in strategy. (We have previously argued that dACC neurons have this property.) In this case, the lack of RPE signals may reflect the specific nature of the gamble task we used: no trial-to-trial learning was required, nor was any trial-to-trial adjustment observed. If so, it would suggest that VS neurons use RPE signals from dopamine neurons to construct a gated adjustment or learning signal that changes based on task context (cf. [85]). Regardless, this finding suggests that the VS does not simply copy the RPE signals of its dopaminergic afferents, and that its responses (at least in this task) are more strongly accounted for by its cortical inputs.

Like reward-based choices, perceptual decisions are traditionally linked to the cerebral cortex. However, recent work by Ding and colleagues clearly demonstrates the role of the striatum in perceptual decisions [102–104]. Indeed, it is striking how reliably the classic perceptual decision correlate can be found in striatal structures [102]. Our results here suggest that similar arguments may apply to the striatum’s role in economic choice, as well. Indeed, we conjecture that many classic prefrontal functions, including choice and executive control, may depend on corticostriatal circuits. Recent work, for example, has demonstrated that the striatum may be involved in executive functions such as planning and cognitive flexibility [105–107]. Taken alongside these findings, our work suggests that the function of the striatum in human decision-making may overlap more with that of the cortex than previously thought.

## Methods

The experimental Methods of this study were identical to those of Strait et al. [15], except that neural activity was recorded from VS rather than from VM.

### Surgical Procedures

Animal procedures were approved by the University Committee on Animal Resources at the University of Rochester and conducted in observance of the Public Health Service’s Guide for the Care and Use of Animals. Two water-restricted male rhesus macaques (*Macaca mulatta*) were trained to perform oculomotor tasks for liquid reward. For each animal, a small prosthesis for maintaining head position was used, and a single Cilux recording chamber with a standard recording grid (Crist Instruments) was placed over the ventral striatum. All recorded neurons were analyzed and reported; no neurons were excluded from analysis. Position was verified by magnetic resonance imaging and a Brainsight system (Rogue Research Inc.). Brainsight is a commercially available system (Rogue Research, Montreal, QC) designed to facilitate intracranial navigation in living animals. The general principle of the system is to combine presurgical placement of magnetically opaque fiducial markers with structural MRI scans, followed by generation of a computerized representation of the cranium and brain. When used by a trained technician (our technician, Marc Mancarella, was formally trained by Brainsight), Brainsight allows placement of electrode tips with ~1 mm precision in the X and Y planes (the Z-plane is affected by standard recording variables, but variability is reduced through careful calibration of the microdrive system). Animals received appropriate analgesics and antibiotics after all procedures. Throughout both behavioral and physiological recording sessions, the chamber was kept sterile with regular antibiotic washes and sealed with sterile caps. No animals were killed and histology was not conducted over the course of this study.

### Recording Site

We defined VS as the coronal planes situated between 28.02 and 20.66 mm rostral to interaural plane, the horizontal planes situated between 0 to 8.01 mm from ventral surface of striatum, and the sagittal planes between 0 to 8.69 mm from medial wall (Figs **2A** and **S4**). Our recordings were made from a central region within this zone, which was selected on a voxel-by-voxel basis and in reference to the Paxinos macaque brain atlas [108]. We confirmed recording sites before each recording session using Brainsight with structural magnetic resonance images taken prior to the experiment. Neuroimaging was performed at the Rochester Center for Brain Imaging, with a Siemens 3T MAGNETOM Trio Tim using 0.5 mm voxels.

### Electrophysiological Techniques

Single electrodes (Frederick Haer & Co., impedance range 0.8 to 4M Ω) were lowered using a microdrive (NAN Instruments) until neuronal waveforms were isolated on a Plexon system (Plexon). Neurons were selected for study solely on the basis of the quality of isolation; we never pre-selected based on task-related response properties or excluded any neurons that surpassed our isolation criteria.

### Eye-Tracking and Reward Delivery

Eye position was sampled at 1,000 Hz by an infrared eye-tracking system (SR Research). The task was controlled by a computer running Matlab (Mathworks) with Psychtoolbox [109] and Eyelink Toolbox [110]. A computer monitor was placed 57 cm from the animal and centered on its eyes (**Fig 1A**). A standard solenoid valve dispensed water rewards.

### Behavioral Task

Monkeys performed a two-option gambling task identical to the one we used in a previous investigation (**Fig 1A**) [15]. Two offers were presented on each trial. Each offer was represented by a rectangle 300 pixels tall and 80 pixels wide (11.35° of visual angle tall and 4.08° of visual angle wide). Options offered either a gamble or a safe (100% probability) bet for liquid reward. Gamble offers were defined by both reward size and probability, which were randomized independent to one another for each trial. Each gamble rectangle had two sections, one red and the other either blue or green. The size of the blue or green portions indicated the probability of winning a medium (165 μL) or large reward (240 μL), respectively (**Fig 1B**). These probabilities were drawn from a uniform distribution between 0% and 100%. Safe offers were entirely gray, and selecting one would result in a small reward (125 μL) 100% of the time.

Offers were separated from the central fixation point by 550 pixels (27.53° of visual angle). The sides of the first and second offer (left or right) were randomized each trial. Each offer appeared for 400 ms followed by a 600 ms empty screen. After the offers were presented one at a time, a central fixation point appeared and the monkey fixated on it for 100 ms. Then both offers appeared simultaneously and the animal indicated its choice by shifting gaze to its preferred offer, maintaining fixation on it for 200 ms. Failure to maintain gaze for 200 ms would return the monkey to a choice state; thus monkeys were free to change their mind if they did so within 200 ms (although they seldom did). Following a successful 200-ms fixation, the gamble was immediately resolved and a liquid reward was delivered. Trials that took more than 7 s were considered inattentive and were excluded from analysis (this removed <1% of trials). Outcomes that yielded rewards were accompanied by a white circle in the center of the chosen offer (see **Fig 1A**). Each trial was followed by an 800-ms inter-trial interval with a blank screen.

Probabilities were drawn from uniform distributions with resolution only limited by the size of the screen’s pixels, which let us present hundreds of unique gambles. Offer reward sizes were selected at random and independent of one another with a 43.75% probability of blue (medium reward) gamble, a 43.75% probability of green (large reward) gambles, and 12.5% probability of safe offers. Note that this means two offers with the same reward size could be presented in the same trial.

### Statistical Methods

PSTHs were constructed by aligning spike rasters to the start of each trial and averaging firing rates across multiple trials. Firing rates were calculated in 20-ms bins, but generally were analyzed in 500 ms epochs. For display, PSTHs were smoothed with a 200-ms running boxcar.

Some statistical tests of neuronal activity were only appropriate when applied to neurons one-at-a-time because of variations in response properties across the population. In such cases, a binomial test was used to determine if a significant portion of individual neurons reached significance on their own, which would allow conclusions about the neural population as a whole.

These animals had previously performed other tasks where the same color hierarchy was maintained (green > blue > gray), but with a different sets of precise amounts. Because of this, we reasoned that the animals would encode reward size ordinally in our task. To account for this, our analyses consistently make use of an ordinal coding of reward size, with gray, blue, and green offers offering 1, 2, and 3 water units, respectively.

The confidence intervals in Figs 2B, 3C and 3D are fit to the data by estimating confidence intervals on regression parameters (betas and intercepts) using a least squares method. The area highlighted in red in each of these figures lies between lines calculated using betas and intercepts from the parameter CI upper and lower bounds.

Fig 4D made use of a decoding analysis. We first separated trials by choice. We required the same number of trials both across neurons and across conditions (offer 1 versus offer 2). Therefore, for each analysis, we first found the lowest number of trials in either of the two conditions across all of the neurons, and used this as the number of trials we would give to our classifier. Although neurons were not recorded simultaneously, we treated them as if they were and grouped trials together across neurons as if they were a single trial. Thus, each of these pseudo-trials was paired with values from each neuron, giving us an n by m matrix (where m is the minimum number of trials in each condition across neurons and n is the number of neurons). The only criterion for grouping trials together was that they fell in the same condition (choose offer 1 or choose offer 2), and thus the trials used differed in terms of other task variables (reward size and probability). We took the mean firing rate of each neuron in each of these trials as input into to a Euclidean nearest-neighbor, leave-one-out classifier. This treats each trial as a point in *n*-dimensional space (where *n* is the number of neurons, and the position in a given dimension was the mean firing rate of one neuron). To classify each trial, we took the mean position of the two groups (choose offer 1 or choose offer 2) excluding the trial to be classified. We then took the Euclidean distance between the current trial and the mean position of the two groups—whichever distance was smaller was the group the trial was classified as.

We performed one analysis to investigate how variance in firing related to variance in choice preference. We started by determining the best-fit curve for firing rate in epoch 1 as a function of the expected value of the first offer. In separate analyses, we fit to a line and to the best-fit second-order polynomial. We then classified each trial based on whether the observed firing rate in epoch 1 was greater or lower than a value predicted by the best-fit function. Finally, we correlated choice with whether firing rate was higher or lower than expected for each trial. We tested for a significant relation within each individual neuron using Pearson’s correlation test of these two sets of variables trial-by-trial. We then repeated this analysis for epoch 2.

## Supporting Information

S1 Fig Likelihood of choosing risky offer instead of a safe one as a function of risky offer expected value. Data are separated for high value (green) and medium value (blue) gambles. Fits are made with a locally weighted scatterplot smoothing (lowess) function. Expected values are calculated in units of ordinal expected value (see Methods).
(TIF)

S2 Fig Effects of seven trial variables on choice (offer 1 versus 2) using a logistic generalized linear model (GLM). Tested variables are: (1) the reward and (2) probability for offer 1, the (3) reward and (4) probability for offer 2, (5) the outcome of the most recent trial (win or choose safe = 1, loss = 0), (6) the previous choice (first = 1, second = 0), and (7) the order of presentation of offers (left first = 1, right first = 0). Error bars in all cases are smaller than the border of the bar, and are therefore not shown.
(TIF)

S3 Fig Unsigned average change in firing rate (+/- 1 standard error) of VS neurons between epoch 1 and the 500 ms preceding epoch 1. Data are separated by the reward size of offer 1. Blue (medium reward size) and green (large reward size) bars only include offers whose expected values were within 5% of the gray (small reward size) offer expected value.
(TIF)

S4 Fig Magnetic resonance image of monkeys B and C. Recordings were made within the nucleus accumbens region of VS (highlighted in orange).
(TIF)

S1 Text Behavioral preference patterns for risky choices and risk preference sensitivity in VS neurons. (DOC)

## Abbreviations

dACC: dorsal anterior cingulate cortex
FSI: fast-spiking interneuron
MSN: medium spiny neuron
OFC: orbitofrontal cortex
PCC: posterior cingulate cortex
RPE: reward prediction error
vmPFC: ventromedial prefrontal cortex
VS: ventral striatum
