# Supplementary figures and images for: Signatures of Value Comparison in Ventral Striatum Neurons
Source: PLoS Biol. 2015 Jun 18;13(6):e1002173. doi: 10.1371/journal.pbio.1002173 (PMC4472856; doi:10.1371/journal.pbio.1002173)

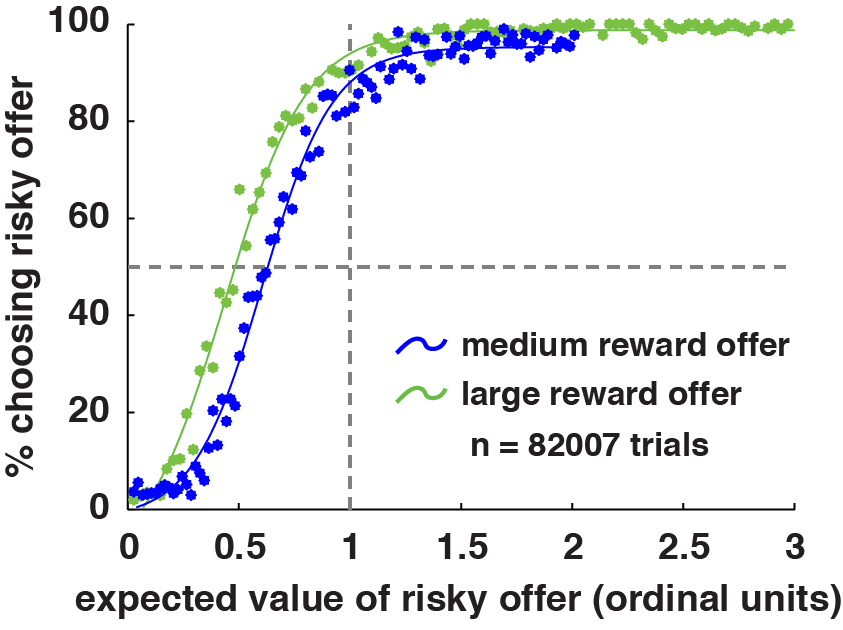

Supplement: S1 Fig — Data are separated for high value (green) and medium value (blue) gambles. Fits are made with a locally weighted scatterplot smoothing (lowess) function. Expected values are calculated in units of ordinal expected value (see Methods). (TIF) [file pbio.1002173.s001.tif]

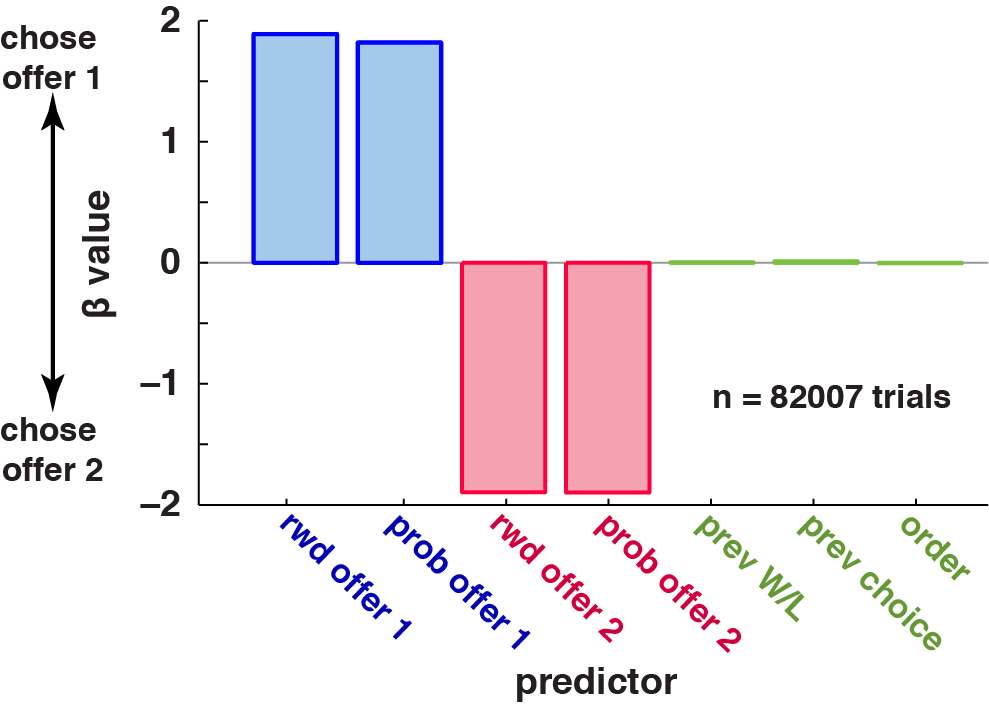

Supplement: S2 Fig — Tested variables are: (1) the reward and (2) probability for offer 1, the (3) reward and (4) probability for offer 2, (5) the outcome of the most recent trial (win or choose safe = 1, loss = 0), (6) the previous choice (first = 1, second = 0), and (7) the order of presentation of offers (left first = 1, right first = 0). Error bars in all cases are smaller than the border of the bar, and are therefore not shown. (TIF) [file pbio.1002173.s002.tif]

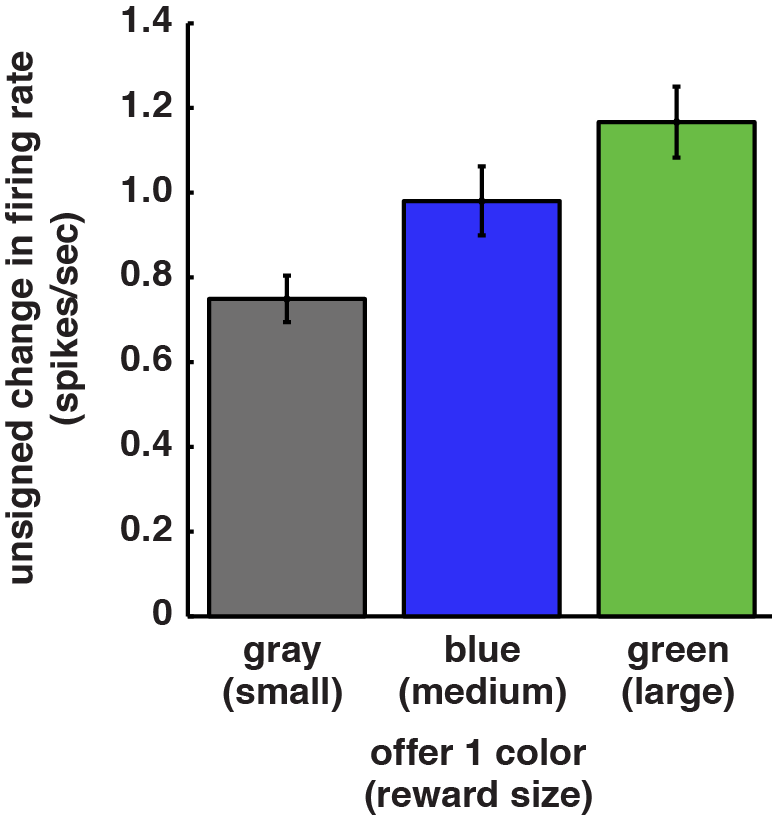

Supplement: S3 Fig — Data are separated by the reward size of offer 1. Blue (medium reward size) and green (large reward size) bars only include offers whose expected values were within 5% of the gray (small reward size) offer expected value. (TIF) [file pbio.1002173.s003.tif]

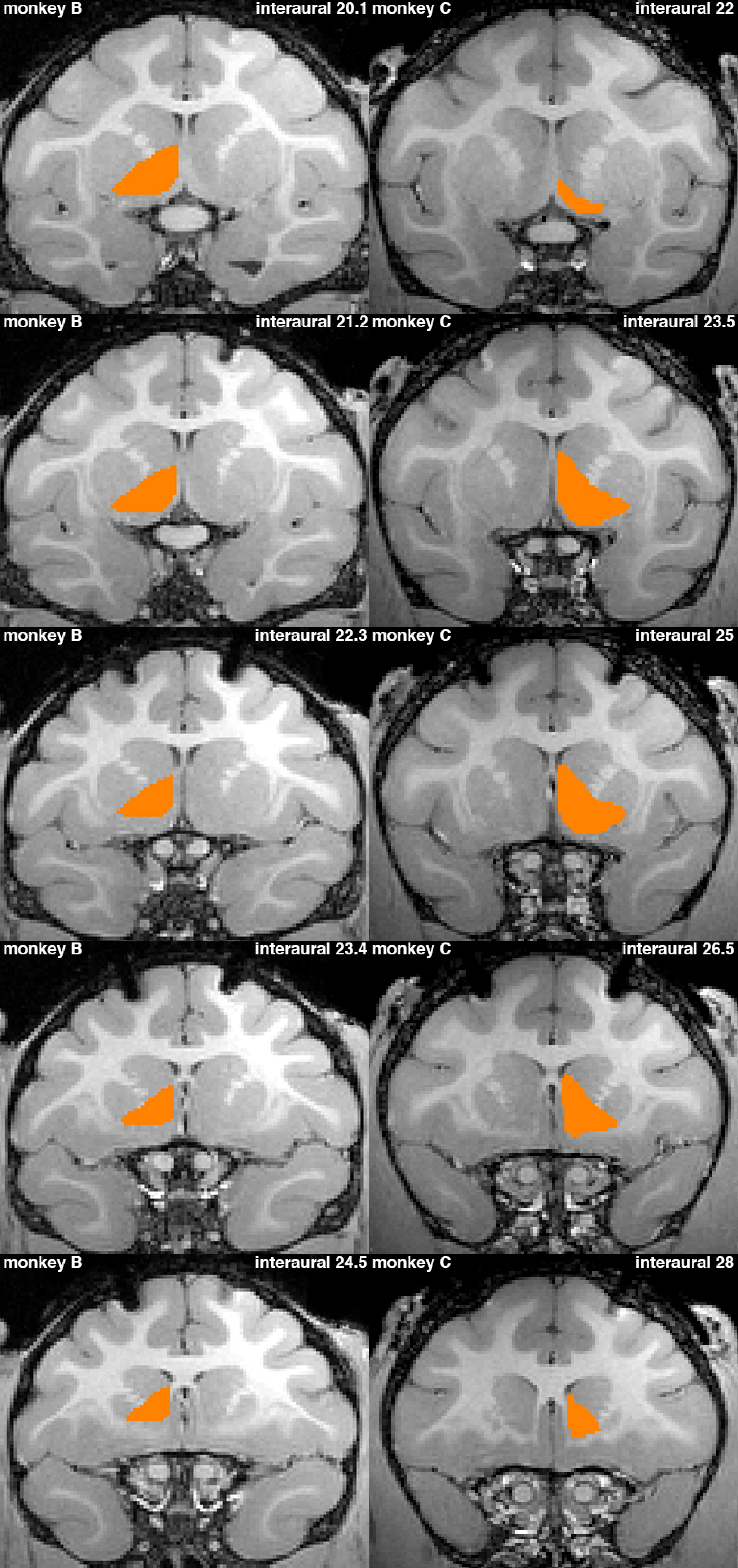

Supplement: S4 Fig — Recordings were made within the nucleus accumbens region of VS (highlighted in orange). (TIF) [file pbio.1002173.s004.tif]
