# Supplementary material for: Signatures of Value Comparison in Ventral Striatum Neurons
Source: PLoS Biol. 2015 Jun 18;13(6):e1002173. doi: 10.1371/journal.pbio.1002173 (PMC4472856; doi:10.1371/journal.pbio.1002173)
Supplement: S1 Text — (DOC) [file pbio.1002173.s005.doc]

**S1 Text**

**Preference patterns for risky choices**

Monkeys chose the offer with the higher *expected value* (i.e. average long-term value associated with the reward) 82.54% of the time (83.02% in subject B, 82.10% in subject C), suggesting that the two monkeys generally understood the task and sought to maximize rewards (n=24711 trials, 11864 in monkey B, 12847 in monkey C).

Monkeys were risk-seeking: they generally preferred risky offers to safe offers when both offers had the same expected values (**S1 Fig.**). This figure averages over data from all sessions, which is then binned by the value of the risky offer (100 bins on the x-axis per reward size). When choosing between EV-matched safe and risky medium reward options monkeys chose the risky option 58% of the time (this bias is significant; binomial test; *P*=0.0042); for EV-matched safe and risky large reward options monkeys chose the risky option 83% of the time (this bias is significant; *P*<0.0001). These findings are consistent with a large body of literature showing risk-seeking in monkeys [1-7], and more broadly with the observation that humans are reliably risk-seeking in repeated risky decisions based on experience rather than description [8].

To further characterize the influence of task variables on monkeys’ choices, we implemented a logistic general linear model with choice (first offer vs. second offer) as a function of 7 regressors (**S2 Fig.**): (*1 and 2*) both reward sizes, (*3 and 4*) both reward probabilities, (*5*) outcome of previous trial (reward vs. no reward), (*6*) choice of previous trial (offer 1 vs. offer 2), and (*7*) side of offer 1 (left vs. right). We found that choice was affected by both reward sizes (offer 1: *β=*1.46; offer 2: *β=*-1.52, both *P<*0.0001) and both probabilities (offer 1: *β=*1.60, offer 2: *β=*-1.60, both *P<*0.0001). Choice was not affected by outcome of previous trial (*β=*-0.01, *P=*0.7717) or by chosen offer order on previous trial (*β=*0.00, *P=*0.97). Moreover, previous outcomes did not affect the side the monkey chose (left offer vs. right offer;  χ^2^=1.13, *P=*0.2878) or previous offer expected value (high vs. low; χ^2^= 1.0723, *P=*0.3004). The order in which options appeared exerted an extremely weak, but measurable, effect on choice (*β=*-0.05, *P=*0.0023). This effect size was only 3% of the next weakest effect, offer 1 reward size. These results indicate that monkeys’ preferences are determined largely by the key economic variables (reward size and probability) and weakly or not at all by variables other than probability and gamble stakes.

**Risk preference sensitivity in VS neurons**

We next examined whether risk-seeking behavior is reflected in VS neurons’ firing rate responses to individual offers. We examined the change in firing rate associated with presentation of three EV-matched classes of offers, safe offers (low reward size, gray bar), low-risk offers (medium reward size, blue/red bar), and high-risk offers (high reward size, green/red bar; this analysis only included low- and high-risk offers with expected values within +/- 4% the expected value of safe offers). We then computed average firing rate response changes for these three offer types. Because our other analyses indicate that neurons in VS show a mix of positive and negative tuning directions for reward, we focused on changes from baseline, regardless of their sign (i.e. we took an absolute value measure). We found larger changes in firing rate for offers with matched expected values and greater subjective values (linear regression; *β*=0.1025, *P*=0.0482; **S3 Fig.**). This result suggests that the value coding in VS neurons is sensitive to risk preferences. This bias was also observed in vmPFC responses to a modestly greater degree (*β*=0.1359, *P*=0.0032; [9]).

**Supplementary References**

1. O'Neill M & Schultz W (2010) Coding of reward risk by orbitofrontal neurons is mostly distinct from coding of reward value. *Neuron* 68:789-800.

2. McCoy AN & Platt ML (2005) Risk-sensitive neurons in macaque posterior cingulate cortex. *Nature neuroscience* 8:1220-1227.

3. Hayden BY, Heilbronner SR, Pearson JM, & Platt ML (2011) Surprise signals in anterior cingulate cortex: neuronal encoding of unsigned reward prediction errors driving adjustment in behavior. *The Journal of Neuroscience* 31:4178-4187.

4. Heilbronner SR & Hayden BY (2013) Contextual factors explain risk-seeking preferences in rhesus monkeys. *Frontiers in neuroscience* 7:7.

5. Monosov IE & Hikosaka O (2013) Selective and graded coding of reward uncertainty by neurons in the primate anterodorsal septal region. *Nature neuroscience* 16:756-762.

6. Seo H & Lee D (2009) Behavioral and neural changes after gains and losses of conditioned reinforcers. *The Journal of Neuroscience* 29:3627-3641.

7. So N & Stuphorn V (2012) Supplementary eye field encodes reward prediction error. *The Journal of Neuroscience* 32:2950-2963.

8. Hertwig R & Erev I (2009) The description-experience gap in risky choice. *Trends in cognitive sciences* 13:517-523.

9. Strait CE, Blanchard TC, & Hayden BY (2014) Reward value comparison via mutual inhibition in ventromedial prefrontal cortex. *Neuron* 82:1357-1366.
